# Suture Materials: Conventional and Stimulatory-Responsive Absorbable Polymers with Biomimetic Function

**DOI:** 10.3390/biomimetics10090590

**Published:** 2025-09-04

**Authors:** Francesco Nappi

**Affiliations:** Department of Cardiac Surgery, Centre Cardiologique du Nord, 93200 Saint-Denis, France; francesconappi2@gmail.com

**Keywords:** suture materials, absorbable polymers, biomimetic function

## Abstract

Suture materials are of pivotal importance in the process of wound healing, as they provide support to growing tissue. The application of suture materials is an intricate process that extends beyond mere closure of skin wounds. Rather, it encompasses a wide range of surgical procedures. It is evident that suture materials possess a high degree of versatility, as evidenced by their application in a broad range of surgical disciplines, including, but not limited to, plastic surgery, neurosurgery, vascular surgery and ocular surgery. Additionally, their application extends to wound treatment and the repair of the musculo-skeletal system and the urogenital tract. This review underscores the pivotal role of sutures in contemporary medicine and surgery. The selection of suture material must be made with the utmost attention to the physical and biological characteristics of the material concerned. The process is characterised by a multifaceted evaluation encompassing the following: first, the assessment of the wound in question; secondly, the healing rate of different tissue types; and thirdly, a thorough appraisal of the patient’s overall physical condition. Advances in suture material technology have given rise to a wider range of sutures, thereby enhancing the existing array of options. Simultaneously, suture needles have undergone a progressive process of technological refinement, resulting in a more comprehensive range of alternatives with a heightened level of precision for specific applications in tissue engineering. Recent experimental investigations have employed an animal model, underpinned by biomechanical analysis. It is evident from the findings of these studies that absorbable sutures fulfil a scaffolding function. The hypothesis concerning the biomimetic function of the materials under investigation was corroborated by the results of biomechanical behaviour and histological examination. This review explores the functionality of both absorbable sutures and novel polymers, investigating their potential application as scaffolding materials within clinical contexts.

## 1. Introduction

Suture material is utilised in a variety of ways during the different surgical procedures. The objective of the procedure is threefold: firstly, to close the fascia; secondly, to stop bleeding; and thirdly, to perform specific tissue anastomoses (intestinal and vascular). Furthermore, the function of suture materials in the process of wound repair is pivotal, as they provide a supportive framework that facilitates tissue healing. For instance, the processes of wound healing and achieving closure can be regarded as complex biological phenomena. This process is characterised by the migration of inflammatory cells, which is followed by the subsequent formation of a new blood vessel network. It is imperative to understand that this sequence of events is critical for the process of wound repair, and that it can be influenced by various factors, including the severity of the injury and the immune response of the individual.

The overall effectiveness of a suture material is contingent upon its physico-mechanical characteristics, its handling properties, and its biocompatible attributes. The optimal material for suture would possess the capacity for universal utilisation, exhibiting optimal handling characteristics, reliable knot security, and tensile strength. It would be required to meet stringent standards of sterility, nonelectrolytic properties, non-ferromagnetic characteristics, non-capillary properties, non-allergenic properties, and non-carcinogenic properties. Additionally, it would be essential to ensure that the material does not serve as a medium for bacterial proliferation. The material must demonstrate resistance to deformation and be readily absorbed by the tissue with minimal alterations to its physical properties. It should be easily sterilised without compromising its structural integrity, and its use should be economical [1,2,3,4]. The search for an ideal suture material remains an ongoing endeavour. The selection of the most suitable suture material for a particular surgical application is a decision that is made by the surgeon, taking into account the specific circumstances of the procedure. The decision regarding the appropriate suture material is contingent upon various factors, including the patient’s age, weight, and overall health status, in addition to the location of the wound. Tissue characteristics, such as thickness, elasticity, healing speed, and scarring propensity, exhibit variability across different anatomical regions. Concurrent pathologies, including but not limited to diabetes, cardiovascular disease, dermatitis, and the utilisation of systemic pharmaceuticals such as steroids, have the potential to influence the healing response. The selection of suture material is influenced by various factors, including the presence of infection and the characteristics of the individual wound. It is incumbent upon surgeons to identify materials that exhibit specific physical characteristics, including but not limited to: a high strength-to-diameter ratio, consistent diameter, sterility, pliability, and optimal tissue acceptance. The performance of these materials should be predictable.

The advent of technological innovations in the domain of suture materials has resulted in a continuous and progressive escalation in sophistication. As a consequence of this, an augmented array of suture options is now available for use. Concurrently, there has been significant advancement in suture needles. This development has resulted in an array of options with heightened specificity for various applications. The evolution of stapling instruments and diverse tissue adhesives has emerged as a substitute for sutures, offering a range of benefits. The process of wound closure is a critical component of medical care, involving the management of wounds to promote healing and prevent complications. Consequently, surgeons currently face the considerable task of comprehending the extensive array of options at their disposal and maintaining currency with the latest advancements in the field.

Selecting the right suture material is vital. This choice is based on understanding the material’s physical and biological properties. It is crucial to carefully evaluate the wound and consider how different tissues heal. Furthermore, it is imperative to undertake a comprehensive evaluation of the patient’s physiological profile to ensure optimal outcomes. It has been demonstrated in a multitude of documented cases that the selection process for candidates has been significantly impacted by the individual preferences and training backgrounds of the surgical team. Furthermore, economic factors have also been observed to exert an influence, with these considerations superseding the utilisation of objective and systematic criteria.

## 2. Understanding the Relationship Between the Properties of Suture Materials and Their Performance

### 2.1. Mechanics

♦ Tensile Strength

According to the United States Pharmacopeia (USP), tensile strength (N/m^2^), defined as the weight required to break a suture divided by the suture’s cross-sectional area, is a crucial metric in the field of suture testing and analysis [5,6,7,8,9,10]. It is imperative to note that the correlation between the requisite tensile force for the rupture of a suture and the suture’s diameter does not adhere to a linear relationship [11,12,13,14,15,16,17,18,19]. The measurement of tensile strength can be accomplished using either dry or wetted surgical sutures. The tensile strength of moist surgical sutures is more clinically relevant, given that sutures are routinely inserted into tissue that contains extracellular bodily fluid. The effective tensile strength is an ancillary metric that appraises the tensile strength of a suture that has been looped and knotted. The effective tensile strength of a suture is contingent upon the material composition of the suture and the configuration of the stitching mechanism [20,21,22,23]. In order to fulfil its designated function, a suture must possess and maintain adequate tensile strength [7,8,10,12].

♦ Tissue Assimilation

The phenomenon of differential grade absorption, which pertains to the body’s capacity to degrade the suture over time, constitutes a hallmark property. It has been demonstrated that the tensile strength of absorbable sutures is reduced as they undergo biodegradation and absorb into the surrounding tissue. Non-absorbable sutures possess a high degree of resistance to degradation; consequently, they are able to retain their tensile properties for extended periods of time. In the context of surgical suture, absorbable sutures are defined as sutures that dissipate the majority of their tensile force within 60 days of being implanted. Conversely, nonabsorbable sutures are characterised by the retention of their tensile force for a period exceeding 60 post-implantation days. Notwithstanding, it should be noted that the reduction in tensile force is not contingent upon the process of dissolution [24,25]. To illustrate this point, one may consider the phenomenon of an absorbable stitch. In such a scenario, the aforementioned stitch may undergo a rapid decline in mechanical properties, determined by tensile forces, whilst concurrently undergoing a gradual process of dissolution. It is an inevitable consequence of the passage of time and exposure to the environment that foreign materials will ultimately experience a certain level of material breakdown. The utilisation of either absorbable or non-absorbable suture types, along with the associated temporal dynamics of their biodegradation, assumes particular significance in the context of suture-related adverse outcomes, including but not limited to wound dehiscence, suture sinus development, and the formation of granulation tissue [22,24,25,26,27,28,29,30].

Absorbable stitches represent a prevalent suturing method in deeper tissue, due to the body’s natural tendency to gradually absorb such materials, thus eliminating the potential for long-term complications [31,32]. Non-absorbable sutures are most frequently employed in the context of surgical interventions involving the skin, where they are utilised to provide structural support during the healing process [33]. In cases of systemic disease or impaired healing, with the requirement for prolonged supportive treatment, the utilisation of non-absorbable sutures in deeper tissues has been demonstrated to be efficacious. A number of factors have been identified as potential causative agents for delayed wound healing, including, but not limited to, diabetes, corticosteroid therapy, nutritional deficiencies, psychological distress, and systemic pathologies. In such instances, the utilisation of non-absorbable sutures is recommended, with their placement being of paramount importance. The configuration of these sutures should be such that their removal can be facilitated upon the completion of the repair process, thereby preventing the occurrence of suture sinus development.

♦ Friction force

The coefficient of friction (μ = F/N) is a quantitative metric that is utilised to evaluate the ease with which a suture can be moved relative to its surroundings. This evaluation is performed across an area of tissue. The passage of the material through the tissue [24,25] is indicative of the quantity of tissue. The occurrence of a traumatic event may result in the formation of a suture. It has been demonstrated that smooth surfaces are associated with a reduced risk of trauma. It has been determined that these elements hold particular relevance in the context of fragile tissular structures, such as those found in the ocular region. Nevertheless, it has been determined that the application of higher levels of tension is a prerequisite for the successful execution of smoother sutures. This is due to the fact that such sutures necessitate the establishment of optimal tissue apposition, whilst concomitantly ensuring that the knot remains securely fastened.

♦ Cross-sectional size

In selecting the appropriate stitch size, it is advisable to opt for the smallest size that will function effectively. This is predicated on the anticipated tightness of the stitch and the robust nature of the tissue [5,6,7,8,9,10]. Thirdly, it is not necessary for the stitch to be more robust than the tissues into which it is sewn. The selection of the most appropriate size is of paramount importance. This approach has been shown to reduce the damage caused by the needle penetrating the tissue, while also ensuring that less harmful extra matter is retained in the area of the cut. The quantity of sutures employed will be contingent on the selected suturing configuration. The objective is to position the skin margins in such a manner that the number of sutures required is minimal, and their diameter is at a minimum.

The most common standard for the recording of suture size is that of the United States Pharmacopeia, which employs a numerical scaling system for the measurement of dimensions. The diameter of the suture is specified in a hierarchical manner, with the numerical value denoting the grade of the suture. For instance, 2-0 denotes a larger diameter than 5-0. Nevertheless, upon the removal of the 0, the suture size undergoes an increase in numerical value, such that the size of the number 3 suture exceeds that of the number 1 suture.

♦ Elasticity

Elasticity is defined as the capacity of a material to undergo deformation when subjected to tensile stress. The process of returning to its initial dimensions after the subsidence of the oedema is referred to as ‘reabsorption’ [24,25]. The property of elasticity is considered a favourable characteristic in the context of material used for surgical sutures. The high elasticity of the stitching enables it to accommodate the stretching of the suture as the wound undergoes oedema. In order to regain the tissue and achieve the desired outcome, it is necessary to cut through the tissue margins. It is to be expected that, as the swelling subsides, the object will return to its normal shape and length. This process is conducive to optimal tissue adhesion during the reparative process.

♦ Knot Security and strength

The knot is considered the most unreliable constituent of a suture [5,6,7,8,9,10]. Its function is to provide a secure hold without exhibiting signs of degradation, such as unravelling or cutting. The force required to cause a knot to slip or break is used to determine its strength [1,2,3,4,24,25]. The relationship between this variable and the measured parameter of the frictional force is direct. The cohesion of a knot is attributable to the frictional forces generated by the application of one knot to an adjacent knot. A stitching material exhibiting a relatively high level of frictional resistance has been shown to enhance knot stability and integrity, thereby ensuring a higher level of security. However, it is also prone to the formation of abrasive forces and the generation of undesirable levels of both resistance and drag during its traversal throughout the layers of bodily tissues [34,35,36,37,38]. The concept of ‘knot security’ is a measure of the ability of a knot to resist undue strain when subjected to repeated manipulation. In essence, this attribute facilitates the execution of a given knot with a minimal number of consecutive knots, thereby ensuring its structural integrity [39,40,41]. In the interest of safety, it is recommended that a knot be secured with a minimum of three. The object in question is characterised by its three millimetre-long extremities, which are designed for the purpose of throwing. In circumstances where sutures are utilised that possess smoother surfaces, it is imperative to employ additional throws in order to ensure that the knot is securely fastened without the risk of undue fraying, severing, or slippage [42,43,44,45].

The concept of effective suture breaking force (SBF) is defined as the maximum tensile force that a suture strand can withstand prior to failure following its knotting. Greater effectiveness in tensile loading has been demonstrated to minimise the risk of wound complications, such as dehiscence. However, it is imperative to ensure that the tensile load capacity of the knotted suture does not exceed the tensile load capacity of the underlying tissue. Regardless of the specific knot arrangement or material employed, the knot itself and the adjacent segment immediately adjacent to the knot are considered the weakest points in a surgical suture. The magnitude of reduction in tensile strength is contingent on the suture material utilised, exhibiting potential variations ranging from a 35% to a 95% decrease, as evidenced by research findings reported in several investigations [20,21,22,23,46,47,48,49]. This phenomenon can be attributed to the inherent dynamics of functional biomechanics, particularly the phenomenon of slip-off of stitching material through the knot and the inevitable stretching of the stitching material that occurs during the formation and tightening of the knot.

It is evident that the knot has been subjected to excessive relative wound tension. Concerns have been raised regarding the potential for suture failure resulting from knot slippage. In an effort to address these concerns, there has been a tendency to tie knots that are tighter than necessary. Nevertheless, if the suture is executed with excessive force, it has the potential to result in localised tissue necrosis, impede fibroblast growth and replication, and lead to inadequate tension equilibrium, which may consequently diminish the structural integrity of the regenerated surgical site.

It is widely acknowledged that surgical knots are characterised by a particularly high degree of consolidation of non-native matter. The material composition of the suture, in conjunction with the magnitude of the knot’s volume, has been demonstrated to directly correlate with the extent of the underlying tissue inflammatory response [46,47]. In the context of wound repair and the promotion of a minimal inflammatory reaction, the primary objective of the surgeon is to ensure that suture knot sizes are minimised while maintaining the optimal tensile stiffness of the entire stitched structure. In such cases, the selection of an alternative suture type may be necessary.

♦ Memory

Memory’ refers to a suture’s ability to retain a consistent straight shape after being removed from its packaging and subjected to stretching. Sutures with greater memory are not pliable and this is something that should be remembered. This results in a suture. This suture has two problems. Firstly, it has inadequate surgical manipulation characteristics. Secondly, it has inadequate secure knot properties [20,21,22,23].

♦ Plasticity

Plasticity and elasticity are interrelated, and this relationship can be understood by looking at how they influence each other. Plasticity is known as the ability of a suture to stretch with wound swelling but remain permanently changed or deformed after the swelling goes down [5,6,7,8,9,10,24,25]. Sutures with high plasticity work in a similar way to elastic sutures. It is imperative to avoid incising the tissue margins in cases of wound tumefaction. Plasticity is generally considered an unfavourable attribute, in contrast to elasticity. When wounds return to normal after swelling, sutures with high plasticity may become excessively slack, which can result in delayed wound closure and the potential for tissue to separate from the underlying layers.

### 2.2. Histology

♦ Tissue Reactivity

Foreign materials are used in suture production, and they may cause an inflammatory tissue reaction, which can hinder the healing process and possibly elevate the probability of complications arising from infection [5,6,7,8,9,10]. The severity and duration of the reaction are contingent on the type, quantity, origins, and method of administration of the suture [10,11,12,13,14,15,16,17,18,19]. The optimal suture material must be nonelectrolytic, noncapillary, nonallergenic, and noncarcinogenic. The implementation of this approach would serve to mitigate the occurrence of adverse tissue responses.

The histology is conditioned by the quality of the material. The quality of the material used for sutures can be defined by three factors: the material’s elasticity, plasticity and memory [5,6,7,8,9,10]. The materials employed are intended to offer both comfort and a natural feel. Silk has become the standard against which other suture materials are compared due to its superior manipulation and simplified knot-tying process [5,6,7,8,9,10,20,21]. The utilisation of flexible sutures, characterised by their capacity to adapt to the anatomical structure, is indicated for vessel ligation and the implementation of continuous sutures. In contrast, sutures exhibiting a lower degree of flexibility, such as those composed of wire, have proven to be inadequate for the aforementioned purposes.

### 2.3. Specific Properties of Suture Materials

♦ Origin and Structural Design

The USP has developed a universal standard for the measurement and categorization of card dimensions, ranging from fine to extra-large [1,2,3,4,5]. The standard of a suture, in accordance with the definition established by the USP, is measured in millimetres and its magnitude is articulated in a numerical scale, ranging from 10 to 0, representing the most minute measurement, to 7, denoting the most substantial measurement. It is widely accepted that zero is the standard mean size for sutures. It has been determined that a decrease in suture calibre is associated with an increase in the number of zeros. For instance, the 10-0 suture, which possesses a finer diameter than the 4-0 suture, is the most diminutive size commercially available. Sutures of larger size are designated with the numerical sequence 1–7. USP 7 is the suture of largest diameter commercially available.

The material employed for the purpose of suturing may be either of a naturally occurring or a synthetic origin. It has been demonstrated that natural fibres, such as surgical gut and silk, elicit a more pronounced pro-inflammatory response in comparison to synthetic materials, including polypropylene. Sutures can be categorised as either single stranded (mono-filament) or multi-stranded (multifilament). Monofilament stiches have been shown to exhibit superior properties in comparison to multifilament sutures, including enhanced strength, reduced tissue drag, and a diminished propensity to facilitate infection. Numerous studies have demonstrated that monofilament sutures are associated with a significantly reduced risk of wound infection [20,21,24,25,28]. Nevertheless, it is acknowledged that the manipulation properties of monofilament stitches are inferior to those of multifilament stitches. The utilisation of multifilament stitches, which can be braided or twisted, has been demonstrated to enhance handling. However, this method also results in augmented tissue reactions and the potential for infection, attributable to the enhanced capillarity [10]. Bacteria have been observed to be capable of evading the process of phagocytosis, a process that occurs when a cell engulfs other cells or particles. This ability is attributed to the ability of bacteria to take refuge in the crevices of braided sutures, where they are able to avoid being recognised and engulfed by the immune system [50,51,52,53].

♦ Capillarity and surface adhesion due to intermolecular forces are critical components

Capillarity is the phenomenon of sutures distributing fluid over their entire length, a property that is contingent on their constituent materials. The inherent limitations in the size and mobility of these two cell types prevent them from accessing the interstices within the fibres, which can result in the prolonged persistence of infection even in the context of effective systems-based delivery of antibiotic medications. Monofilaments demonstrate an absence of capillarity, while multifilament stitches exhibit varying degrees of capillarity, a property that is contingent on their chemical composition. It has been demonstrated that a braided nylon suture has the capacity to harbour a quantity of organisms that is up to three times greater than that which would be observed in a non-braided suture of the same material. The utilisation of bacteria as a monofilament nylon suture is a novel approach in the field of surgical innovation [54,55,56,57]. Braided polyester has been shown to exhibit capillarity and the potential to promote the spread of infection, in contrast to braided silk, which, when coated with wax, does not demonstrate such properties [55,56,58,59,60]. The coating of sutures with Teflon, silicone, wax, paraffin wax, and calcium stearate has been demonstrated to reduce both capillarity and the suture’s coefficient of static friction [61,62,63,64].

♦ Fluid Uptake

It is important to note that fluid uptake is distinct from capillarity. Nonetheless, both processes have the potential to contribute to an increased risk of bacterial contamination and its subsequent propagation. In a professional setting, chromic and plain gut sutures are considered to have the greatest fluid absorption potential. These sutures, which are classified as monofilament, are distinguished by their lack of capacity for capillarity [55,56,58,59,60]. The chemical composition and physical configuration of a stitch are pivotal in dictating fluid uptake, with the chemical composition proving to be a predominant influencing factor [28,33]. It has been demonstrated that natural stitches have a superior ability to facilitate fluid uptake when compared with synthetic alternatives, which possess a higher degree of hydrophobicity. However, in general, multifilament stitches tend to show higher levels of fluid uptake in comparison to monofilament stitches [20,21].

## 3. Sutures: An Overview

The material used for suturing can be categorised according to its composition and structure as follows: it may be resorbable or non-resorbable, natural or synthetic, monofilament or multifilament. Resorbable sutures are materials that are characterised by their ability to undergo biodegradation, a process that typically occurs within a period of 60 days following their implantation into the body. This process of resorption is accompanied by a significant reduction in tensile strength, thereby leading to the degradation of the sutures [65,66,67,68,69]. The uptake of naturally occurring biomaterials is achieved through the process of enzymatic degradation, while the uptake of newly developed synthetic biodegradable stitches is facilitated by non-enzymatic hydrolysis. In the course of this process, the polymers are cleaved directly into monomers by water, and the monomers are further degraded to yield carbon dioxide and water [70,71,72]. The utilisation of novel polymeric sutures offers a distinct benefit, namely their resistance to the impact of inflammation or infection on their rate of nonenzymatic hydrolysis during the healing process [73,74,75,76,77].

Material used for suturing which is not resorbable is not subject to degradation to any significant extent following its implantation. The utilisation of these sutures is typically indicated in circumstances where there is a necessity for prolonged support. The prevailing tendency is towards the utilisation of synthetic materials, which exhibit a reduced reactivity in comparison to natural fibres. In comparison to synthetic fibres, natural fibres appear to elicit a more substantial and intense pro-inflammatory response.

The utilisation of resorbable sutures has been demonstrated to be advantageous in circumstances where sustained suture reinforcement is not necessary, or when the removal of sutures is anticipated to be accompanied by discomfort or complications due to their anatomical position. In the context of infection, monofilament non-absorbable sutures are generally considered to be the preferred choice over multifilament varieties. The possibility of complications arising, particularly in the context of infection development, is a salient concern. The phenomenon of capillarity, as observed in multifilament sutures, has been demonstrated to enhance the attraction and retention of bacteria. This, in turn, has been shown to elevate the risk of infection, and the subsequent development of draining sinus tracts from the wound. Table 1 shows the features and uses of different types of suture materials.

### Resorbable Suture Materials

♦ Polydioxanone

**Figure 1:**
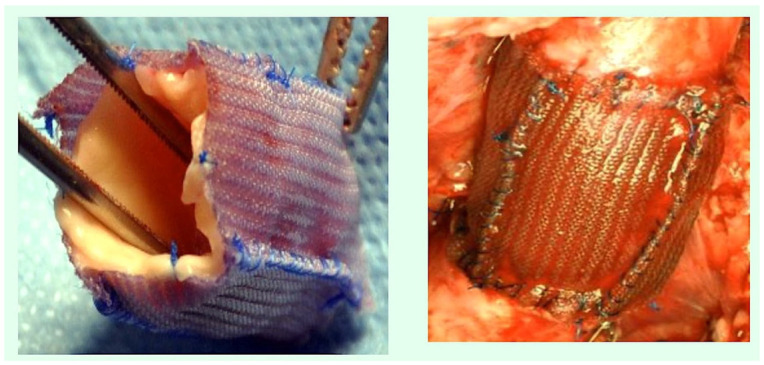
The pulmonary autograft is reinforced with an external resorbable reinforcement layer. Polydioxanone is utilised as an external scaffold for the purpose of providing external support.

Polydioxanone (PDS II) is a synthetically produced, resorbable monofilament surgical suture composed of a polymer of polidioxanone. With the objective of replicating the paediatric clinical scenario, an experimental model of transposition of the pulmonary trunk as an autograft in the aortic position has been developed and performed using cardiopulmonary bypass in 20 growing lambs has been described. Technical and anatomical issues necessitated the reimplantation of the PA in the descending aorta, with the pulmonary trunk being replaced by a homograft from another lamb of the same age and weight. The pulmonary autograft was reinforced by the utilisation of resorbable poly (dioxanone) as a form of scaffolding support [78,79,80,81,82,83,84,85] (Figure 1).

**Figure 2:**
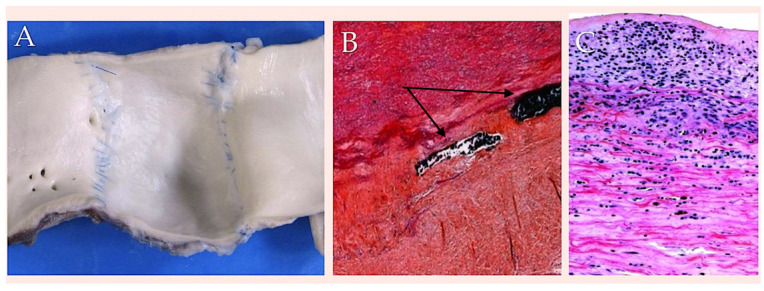
The following image presents a macroscopic and microscopic view of a reinforced explanted pulmonary autograft at six months post-surgery. The graft has been reinforced with a biodegradable mesh. (**A**) The tunica intima has undergone no disruption and is otherwise free from abnormality; (**B**) The tunica media displays normal thickness and is devoid of any disruption. The presence of PDS remnants is indicated here by the black arrow. (**C**) The tunica intima exhibits intimal hyperplasia. Abbreviations; PDS, polydioxanone.

The synthetic absorbable suture under scrutiny has been found to exhibit greater initial tensile strength than polyglycolic acid and polyglactin 910. However, it has also been determined to possess the most deficient degree of knot reliability in comparison to the other synthetic resorbable sutures [86]. The suture exhibited a retention of 74% of its tensile strength following a two-week period, 50% after four weeks, and 25% after a six-week interval. Evidence suggests that the rate of absorption is minimal by 90 days. Furthermore, it is not until 6–7 months following implantation that complete absorption is achieved, whether the material is used as a surgical stitch or as a scaffold for external reinforcement [20,21,78,79,80,81,82,83,84,85,86,87,88] (Figure 2).

**Figure 3:**
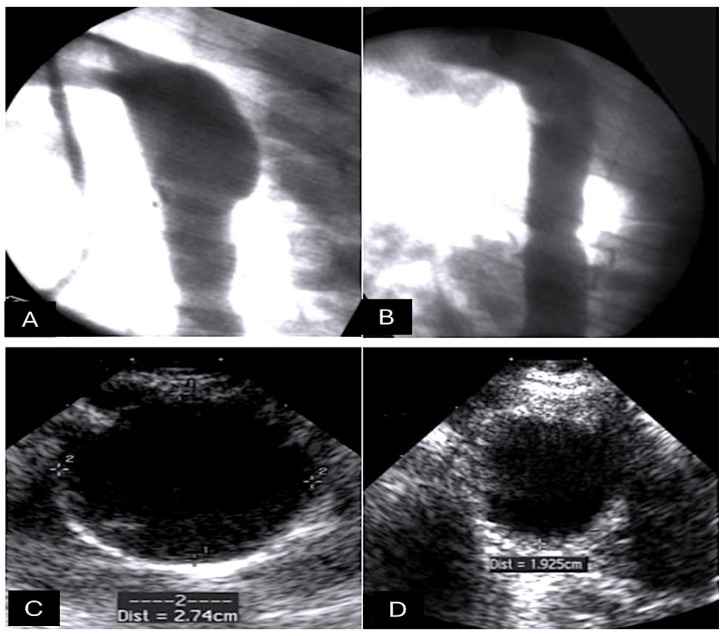
As demonstrated by the figure, the representative angiography study encompasses the following groups: (**A**) control and (**B**) resorbable suture materials comprising polydioxanone and polyglactin. Representative transesophageal images from the following groups are shown: (**C**) Control and (**D**) Resorbable.

The capacity of this synthetic absorbable suture to retain its structural integrity post-implantation constitutes a distinct advantage over competing products in the marketplace. Its use is indicated in circumstances where an extended approximation of tissues is necessitated, with a duration of up to six weeks and under conditions of tension [35]. PDS II is characterised by increased rigidity and a more challenging handling profile in comparison to Dexon and Vicryl, yet it exhibits remarkable ease of passage through tissue. The development of PDS II was initiated with the objective of enhancing the handling characteristics of the original form. It exhibits minimal reactive properties and preserves the structural integrity of infected tissues and urine, making it a frequently employed agent in bladder surgery [36]. Its prolonged retention time has the potential to act as a focal point for the development of urinary calculi in patients with a history of such conditions. Notwithstanding, its compatibility with a broad spectrum of tissues renders it a versatile solution [1,2]. In an experimental animal model, the resorbable matrix sutures were shown to be safe and effective when used for the external reinforcement of the vascular graft. On day 1, the angiographic evaluation of the vessel wall showed minimal distension. Over the next 6 months, however, there was a slight but significant increase in diameter, and the team found no evidence of aneurysmal degeneration. There was no statistically significant difference found either with polidioxanone or polyglactin 910 when used as external support, and the mean diameter was found to be 28 +/− 2 mm. The echocardiographic examination confirmed these variations. The pulmonary autografts in groups using resorbable suture material showed minimal distension on day 1, followed by a slight but significant increase in diameter over 6 months. A mean diameter of 28 mm (±0.85 mm) was found, and no difference was detected among the various resorbable materials, which included polydioxanone and polyglactine 910 [81,83] (Figure 3).

♦ Polyglactin 910 (coated Vicryl, Vicryl Rapide, and coated Vicryl Plus)

**Figure 4:**
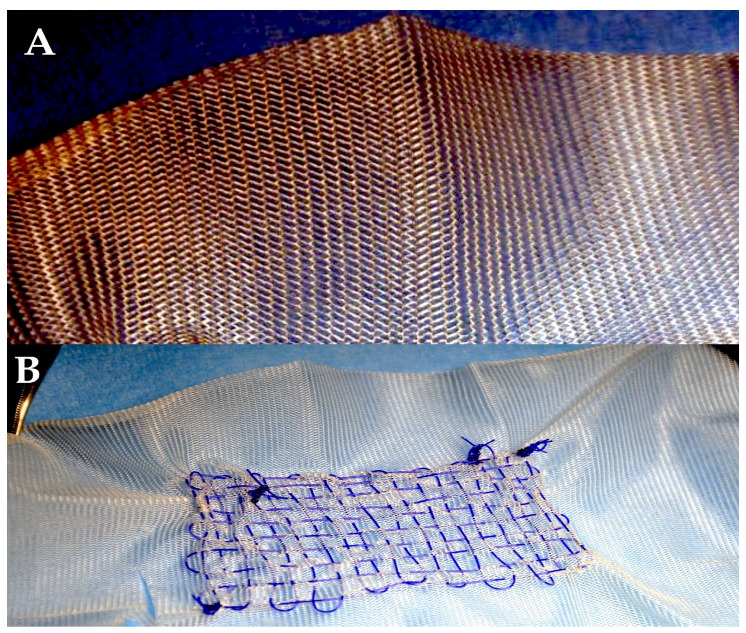
The following illustration is to be used to depict the absorbable materials. (**A**) Polyglactin 910 mesh (Vicryl). (**B**) Polydioxanone suture, assembled with Vicryl (B).

The polyglactin (PG) suture was the secondary synthetic resorbable stitch to be introduced, subsequent to the Dexon stitch (polyglycolic acid). Vicryl is a braided synthetic stitch that is composed of polyglactin 910, a copolymer of L-lactide (10%) and glycolide (90%), and is additionally covered with calcium stearate [89]. Polyglactine can be used to provide structural support as a standalone suture or as a component of a scaffold, particularly for stabilising arteries. It can also be combined with polydioxanone, a material used in surgical adhesives [78,79,80,81] (Figure 4).

**Figure 5:**
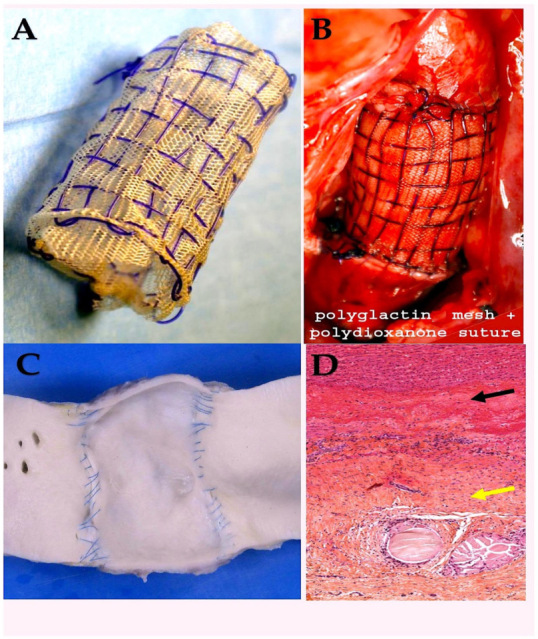
The accompanying illustration delineates the utilisation of absorbable materials, which are employed for the purpose of either mesh or stitching. (**A**) A composite scaffold is constituted for the purpose of external reinforcement. (**B**) The composite scaffold, composed of polyglactin 910 mesh and polydioxanone suture, serves as an external reinforcing structure that mimics the biological properties of the suture. (**C**) At the six-month stage, the tunica intima of the vessel wall exhibited an intact structure, devoid of any disruption or inflammatory response. (**D**) The pathological examination revealed no abnormalities in thickness, with the tunica media (yellow arrow) and tunica intima (black arrow) exhibiting no signs of disruption.

Vicryl is subject to sterilisation by ethylene oxide. The product is available in both transparent and dyed forms. Vicryl has been shown to exhibit a number of similarities with Dexon in terms of its chemical composition and physical properties. As a material, it demonstrates a higher level of initial resistance to tensile forces than surgical gut, yet its strength is not significantly superior to that of Dexon. This discrepancy is considered to be of negligible clinical significance. Vicryl demonstrated a retention percentage of tensile strength ranging from 50% to 65% at the two-week mark, however by the three-week point, no residual strength could be detected [20,21,78,79,80,81]. The substance in question has been shown to elicit a minimal inflammatory reaction, with complete absorption occurring by way of hydrolysis. The duration of this process is between 60 and 90 days. The product exhibits commendable handling characteristics and an optimal size-to-strength ratio, rendering it well-suited for utilisation in a range of tissues, including those afflicted with infection [20,21,78,79,80,81]. The product is available in a range of dimensions. Furthermore, it has been utilised as resorbable scaffold in experimental models on animals [66,78,79,80,81]. In the experimental setting, all pulmonary artery specimens fortified with a bioresorbable mesh exhibited a smooth endoluminal surface, independent of the material used. This finding aligns with that observed in the aorta (Figure 5). In the cohort of subjects assigned to undergo treatment with the Polyglactin, it was found that the thickness of the pulmonary artery was within the bounds of normalcy. From a histological perspective the presence of intimal hyperplasia was once again observed. The tunica media was found to be intact, with the remnants of the slowly resorbable tissue. On the contrary, PDS sutures were frequently observed in the adventitia (Figure 2B). Masson’s trichrome staining (obtained from Sigma-Aldrich, St. Louis, MI, USA) revealed the preservation of the endothelium and the reorganisation of the tunica media. Directly beneath the intima, smooth muscle cells were observed to be intertwined with collagen fibres; deeper layers revealed collagen bundles to be intricately interwoven with elastic fibres, forming a substantial and highly organised layer of concentric lamellae. The tunica adventitia was formed by loose connective tissue with adipocytes (Figure 5D) [78,79,80,81].

Vicryl Rapide is a synthetic suture made of multifilament material that is able to be gradually absorbed by the body over time. The suture is composed of polyglactin 910, a synthetic material that undergoes partial hydrolysis in a buffered solution, followed by sterilisation through gamma ray exposure. The processing of the substance in question has been demonstrated to enhance its absorption [20,21]. The material in question has been shown to possess approximately 66% of the original tensile stress of similarly treated Vicryl [85,86,87,88]. As evidenced by numerous studies in the field, it has been demonstrated that the material in question exhibits a significantly faster loss of tensile strength in comparison with Vicryl. Consequently, it is employed in the following manner. The oral cavity is subject to a process of sloughing, which commences within a period of 7–10 days [1,2,3]. In other tissues, a loss of strength is observed within 7–14 days, and complete uptake is achieved by 21 days. The design of the device was conceived with the intention of replicating the functional characteristics of surgical gut without instigating the associated postoperative inflammatory response. The material is particularly well-suited for tissues that exhibit accelerated tensile strength gain, such as those found in the gastrointestinal and urinary tracts, where long-term structural support is not a prerequisite [1,2,3].

Coated Vicryl Plus is a suture that has been treated with a coating of the antimicrobial triclosan. It is hypothesised that this inhibits bacterial colonisation of the braided suture, thereby minimising the pain associated with subclinical infection [20,21,24,25]. The treatment is frequently employed in paediatric surgery. It has been demonstrated that the antibacterial properties of the substance in question have the capacity to mitigate the discomfort experienced by infants.

**Figure 6:**
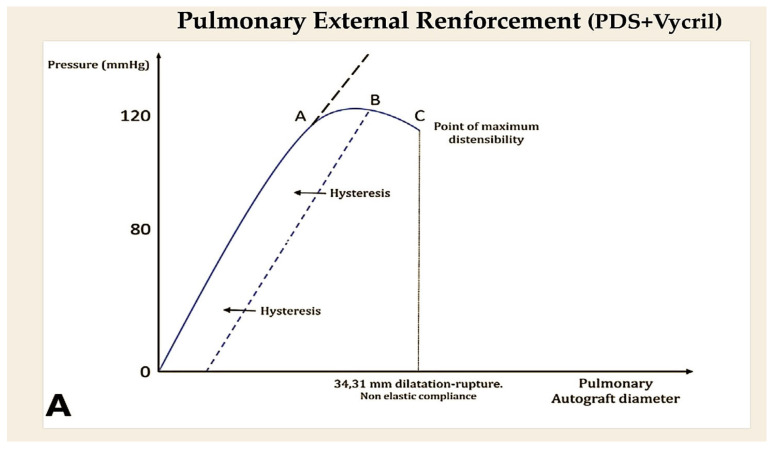

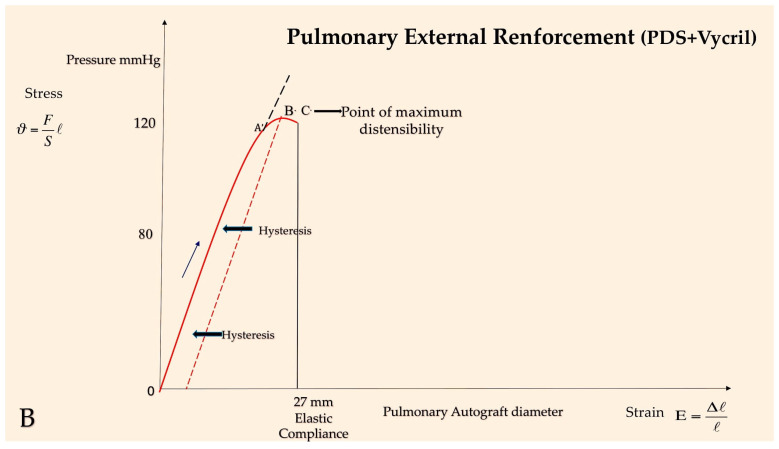
The model under consideration is a simple mathematical one, with the relevant curves being fitted based on the neo-Hookean hyperelastic behaviour of the vessel. (**A**) In the absence of reinforcement. The graph illustrates this point. A = term denoting the elastic behaviour; B = non-elastic behaviour; C = maximum distensibility. (**B**) The product incorporates a resorbable reinforcement element. PDS, in conjunction with Vycril, is utilised as a constituent of a crosslinked bioresorbable external reinforcement. This combination has the capacity to modify the behaviour of distensible materials in terms of their curvature. The promotion of elastic remodelling by PDS plus Vycril has been demonstrated to influence the elastic region of the graph. This has been shown to result in a shift in points A, B, and C, which in turn has led to a reduction in the critical area determined by the fall of the curve. Abbreviation; PDS, polydioxanone.

A number of studies have employed the use of resorbable suture composed of PDSII/PG, utilised as a scaffolding apparatus for the external support of vascular walls [81,82,83,84,85,86,87,88]. These studies have indicated an occurrence of initial dilation of the pulmonary artery positioned in aortic position (18 mm +/− 1 mm) and a slight increase in diameter at the six-month mark (27 mm +/− 2 mm), with an overall augmentation of 42% and an indexed ratio of 1.42. Furthermore, the reference aorta diameter exhibited an increase of 34%. Based on the analysis of curve slopes, it was observed that only the pulmonary artery with resorbable reinforcement material demonstrated a comparable behaviour to the normal aorta in the growing lamb (slope 1.4% and 1%, respectively) (Figure 6A,B) [83,84,85,86,87].

The biomechanics analysis [85,86,87] was corroborated by the presence of histological evidence, which demonstrated the occurrence of compensatory intimal hyperplasia in the reinforced vessel wall [81,82,83,84]. The vessel wall was reinforced using PDS or Vycryl, alone or in combination with a mesh and suture (Figure 4 and Figure 5). The vessel wall was reinforced with external resorbable suture support, a procedure which has been shown to be an effective treatment in such cases. Furthermore, the absence of intimal tears and remnants is also notable. The presence of slowly resorbable PDS sutures was detected in the adventitia (Figure 2B). It is worthy of note that Masson’s trichrome stain revealed a rearrangement of the tunica media, which manifested as a dense fibrous network primarily composed of elastin, organised in eccentric lamellae. This observation suggests an occurrence of elastic remodelling in the reinforced pulmonary autograft (see Figure 5D). The presence of specific staining indicated an increase in the content of elastin in comparison with the control group. Elastin was observed to be organised in a compact layer at the level of the vessel’s “elastic zone”. Concurrently, the reinforced pulmonary artery exhibited an elevated level of metalloproteinase-9 in comparison with the control group. This finding indicates an underlying process of extracellular matrix remodelling, thereby confirming the impression of elastic remodelling and arterialisation. This finding corroborates the observations of elastic remodelling and arterialisation. Furthermore, the presence of inflammatory infiltrate was minimal, with only sporadic macrophages or monocytes observed within the reinforced arterial wall. This finding indicates that the biomaterial did not elicit an excessive inflammatory foreign-body reaction over time [84,87].

♦ Poliglecaprone 25 (Monocryl)

Poliglecaprone is a synthetically produced monofilament that is capable of being absorbed by the body. The substance under discussion is constituted of a copolymer of glycolide and e-caprolactone. The primary function of the applied lubricant coating is to minimise the coefficient of frictional force. The material is available in two forms: one that has undergone the dyeing process, and one that has not. The material is considered to be among the most resorbable of its kind, a property that enables the utilisation of suture sizes that are one to two magnitudes smaller than would typically be selected [24,25,89]. Nevertheless, it has been observed to undergo a rapid decline in strength post-implantation [20,21,81,82,87]. It is evident that dyed monocryl exhibits 30–40% retained tensile force after a period of two weeks, while undyed monocryl demonstrates a significantly lower retaining capacity of 25% at the same time frame. By the third week, there is no residual strength, and absorption is by hydrolysis, which is completed within 90–120 days [20,21]. Monocryl has been shown to elicit negligible tissue response, exhibit optimal knot stability, and provide superior manipulation properties due to its flexibility and minimal memory. In comparison to other synthetic resorbable stitches, Monocryl demonstrates reduced rigidity and a diminished tendency to change shape. In comparison with Vicryl Rapide, when employed in a subcuticular closure, Monocryl resulted in scars that were significantly smaller, exhibited reduced reactivity and exhibited a reduced tendency to produce hypertrophic scar formation [90,91]. Its utilisation is endorsed for a range of soft tissue closure procedures, encompassing instances where subcutaneous approximation is required.

♦ Polyglycolic acid (Dexon, Dexon II)

Polyglycolic acid was the pioneering commercially available resorbable synthetically derived stitch. The suture under scrutiny is a braided synthetic resorbable material manufactured from a synthetically derived homopolymer of glycolic (hydroacetic) acid [20,21,81,82,87]. Dexon II has been coated with polycaprolate, a material that has been proven to enhance the coating’s handling characteristics by reducing the coefficient of friction. Initially, polyglycolic acid demonstrates greater strength than surgical gut, but in comparison to other synthetic absorbable sutures, it exhibits reduced tensile strength. The material demonstrated 89% of its tensile strength after seven days, 63% after fourteen days, and 17% after twenty-one days. It was fully absorbed within the range of ninety to one hundred and twenty days. In comparison to Vicryl, Dexon exhibited a faster loss of its functional properties and a lower knot-breaking strength [20,21].

The Dexon is subject to hydrolysis as opposed to being subject to enzymatic breakdown, which results in reduced tissue reaction and delayed absorption when compared with surgical gut. The final products of this process, namely the degradation products, have been found to be deficient in terms of their capacity to provide the essential growth materials required by most bacterial species [92,93,94]. The utilisation of polyglycolic acid in the oral cavity or in the presence of infected urine has been documented to be inadvisable. This is attributable to its elevated pH which accelerates its rate of degradation [92,93,94]. Conversely, its use is deemed permissible under other circumstances. The use of the device [95,96,97] is indicated when a long-term approximation of the tissue under stress is not required. Illustrative cases include intestinal anastomosis and caesarean surgery [98,99,100,101].

♦ Polyglycolide–trimethylene carbosnate (Maxon)

Maxon is defined as a synthetically produced, resorbable, monofilament copolymer of glycolide and trimethylene carbonate. In comparison with polyglactin 910 and polypropylene, it is characterised by superior handling properties. Nevertheless, it is regarded as more challenging to manipulate than polydioxanone [102,103,104,105]. Recent research has indicated that Maxon exhibits superior knot reliability in comparison with PDS II [102]. It has been demonstrated that, upon implantation, this suture exhibits superior initial tensile resistance when compared to the majority of other resorbable alternatives. However, subsequent to implantation, a decline in tensile strength is observed, with values of 81% at fourteen post-implantation days, 59% at twenty-eight days, and 30% at forty-two days. These values approximate the 30% decrease exhibited by PDS II at the same time points. (10,106) On average, Maxon exhibits a retention time of tensile strength ranging between 42 and 92 days, in comparison to the 64–80 day retention time exhibited by the PDS II [20,21]. Furthermore, the substance is absorbed through a process known as ‘hydrolysis’. As with other synthetic absorbable sutures, and similarly with PDS II, loss is an inherent possibility. Tensile strength is observed to manifest well in advance of a substantial decline in mass. It is estimated that the complete absorption process is accomplished within a period of six months [24,25].

♦ Caprosyn

The suture material under consideration here is Caprosyn. This is characterised by its monofilament structure and its synthetic nature. It is resorbable and is produced from polyglytone 6211 synthetic polyester, which is composed of glycolide, caprolactone, trimethylene carbonate, and lactide [106]. It is a material that has been developed relatively recently and it is subject to rapid uptake by the body. Caprosyn has been demonstrated to maintain a minimum of 50–60% of knot integrity at five post-implantation days, and a minimum of 20–30% at ten post-implantation days. It has been demonstrated that the entirety of its tensile strength is lost by twenty-one days following implantation, with complete absorption occurring by fifty-six days. A parallel has been drawn between Caprosyn, chromic gut and new synthetic monofilament absorbable suture [107,108]. Caprosyn has been demonstrated to exhibit superior tensile properties in comparison to chromic gut, while maintaining equivalent resilience against wound disruption. Caprosyn has been demonstrated to exhibit superior handling properties, enhanced tissue apposition, and enhanced performance in terms of knotting stability. It has been demonstrated that both Caprosyn and Chromic Gut are subject to similar rates of bioavailability; however, Caprosyn has been shown to exhibit superior anti-infective properties. The product has been specifically formulated for use in plastic surgical procedures, obstetrics and gynaecology, and urology, where the advantages of its fast-acting properties and reduced scarring potential may be particularly relevant [106].

♦ Polyhydroxyalkanoates (PHAs)

Biodegradable polymers of microbial origin are known as polyhydroxyalkanoates (PHAs). They are highly biocompatible and environmentally friendly, making them attractive components for elastic medical devices in the field of biomedical engineering [109]. Out of all the PHAs that are currently available, only 4-hydroxybutyrate (4HB) polymers have any clinical applications at all. These are mainly used in sutures and cardiovascular stents [110]. Increases in the 4HB fraction [111] result in greater extensibility of poly(3-hydroxybutyrate-co-4-hydroxybutyrate) (P(3HB-co-4HB)), making it a promising material for extensible medical devices. Nevertheless, the evolution of P(3HB-co-4HB) has been impeded by the ensuing variables: (i) The incapacity to synthesise high-molecular-weight P(3HB-co-4HB) [112] through chemical means; (ii) The scarcity of facilities for conducting microbial synthesis, (iii) The technical complexity of culturing and purifying stable high-molecular-weight copolymers while preserving a constant copolymerization ratio, (iv) The difficulties associated with upscaling bioprocessing, and (v) The challenges of spinning the purified copolymers.

A comparison is made between the physical properties of P(3HB-co-4HB) (United States Pharmacopeia [USP] 2-0, 4HB content = 16 mol%) and those of other resorbable sutures [113]. P(3HB-co-4HB) was compared to Monomax^®^ (USP 2-0) and was found to have a tensile strength that was 50% lower (167 MPa), a Young’s modulus that was around 50% lower (261 MPa), and a breaking elongation that was two times higher (113%). This meant that it was supple and extensible. A substantial residual strain remained after 100% extension deformation. This was 15%. Plastic deformation exceeded its elastic limit. It did not break, unlike other sutures. In the cyclic test, the material was extended by 50% before being allowed to recover. At this point, the Monomax^®^ material had a residual strain of 20%, while the newly developed suture had a residual strain of 7%. This meant that the recoveries were 80% and 93%, respectively. A knot was formed using the P(3HB-co-4HB) monofilament suture (USP 2.5-0). The suture’s circumference is smaller than PDS^®^ II’s (4.03 mm vs. 4.77 mm), its knot area is smaller too (0.843 mm^2^ vs. 1.20 mm^2^), and its knot area to thread diameter ratio is lower (3.28 vs. 7.32). Nevertheless, the former possesses a greater thread size (0.256 mm as opposed to 0.163 mm) and as a consequence, a considerably reduced knot diameter (*p* = 0.0079) [113].

Immersion in Dulbecco’s buffer for 12 weeks caused a decrease in the initial linear tensile strength of the P(3HB-co-4HB) sutures from 161 to 104.6 MPa (i.e., by 35.0%). The fractional weight-average molecular weight (Mw) exhibits a decrease. This is a function of immersion time. The value before immersion is 320,000. The Da is 100%. There is a 50% decrease after 16 weeks. Additionally, the P(3HB-co-4HB) suture exhibited elevated elasticity and notable extensibility at break (160%, compared to the initial measurement of 240%) [113].

Sutures made of P(3HB-co-4HB) were used to close the wounds of the rats in the animal model that were evaluated (X). The strength of these sutures was then tested. The results of the tests, which were performed in rats and in a laboratory, were similar. At week 4, the strength was about 77% and at week 16 it was about 63%. The first linear tensile strength was maintained at 50% for 26 weeks. This is also known as the 50% tensile strength retention time. A 50% decrease in Mw is shown with an increase in implantation time, 16 weeks after the initial implantation. Moreover, the P(3HB-co-4HB) suture’s extension at break persisted at around 200%, maintaining this level 26 weeks post-implantation [113].

## 4. Discussion

The biomimetic properties of resorbable suture have led to the hypothesis that these constituents, particularly polydiaxone and polyglactine, may be utilised as standalone or combined scaffolding to reinforce the vessel wall. For instance, seminal studies on the external support of pulmonary autograft have demonstrated increased stabilisation and solidity of the neoaortic root, thereby preventing its dilation due to systemic pressure. It has been documented that prior experimentation has been conducted with a polyester non-resorbable material, featuring an artificial aortic root configuration (Valsalva graft), which was utilised as an external reinforcement for the pulmonary artery [114,115]. The present approach was adopted with the objective of averting neoaortic root dilatation and the dynamic function of Valsalva sinuses. This technique has been shown to result in the most physiologically optimal pressure and flow patterns in the pulmonary artery in comparison with straight polyester grafts [116,117,118,119]. In this case, the pulmonary artery was encased in a straight polyester non-absorbable prosthetic graft, which resulted in a significant impairment to its pulsatility and compliance. Furthermore, earlier research has indicated that the Dacron graft and other synthetic polyesters have the capacity to markedly compromise aortic compliance when utilised as a vascular replacement, as evidenced in study [120]. Additionally, these materials have been observed to trigger a robust inflammatory response, resulting in substantial damage to the vessel wall when employed as a pulmonary artery reinforcement, as outlined in study [82,83]. The utilisation of resorbable polymers, such as PDSII and Vicryl, whether as a standalone material or as part of a composite scaffold, has been demonstrated to impede pulmonary artery dilatation. The resorption of biomimetic resorbable polymers has been demonstrated to promote a connective remodelling of the vessel wall, resulting in a “neovessel” with increased elastin content and therefore potentially improved biomechanical properties.

Promising novel research is devoted to the study of nickel and nickel-chitosan nanowires [121,122]. Glucose sensors play a vital role in the everyday healthcare needs of diabetic patients, helping to ensure they can manage their condition and live long, healthy lives. However, the cost and reliability of glucose sensors, particularly their standard functionalisation with expensive and environmentally sensitive enzymes, remains a challenge. A method for the fabrication of nickel nanowire arrays (NWAs) coated with a thin layer of chitosan for the non-enzymatic detection of glucose was reported, and it was found that this method was effective. The method involves the electrodeposition of nickel into anodic aluminium oxide (AAO) templates, which is then followed by a special process for coating the templates with chitosan. Electron microscopy, Raman spectroscopy and electrochemical techniques were used to characterise the nickel and nickel chitosan NWAs. Electrochemical analysis involving cyclic voltammetry and chronoamperometry revealed that the addition of a chitosan coating to the sensor greatly improved its ability to detect glucose, even when there were interfering substances present. The sensor’s sensitivity was improved by 46.39% by the coating, and its linear detection range was expanded from 3.85 mM to 4.37 mM. The chitosan coating also retained these characteristics after physiologically accurate sample testing and prevented biofouling after testing with proteins. This straightforward and durable glucose sensor paves the way for the creation of glucose sensors with a high linear range that do not need to be functionalised with traditional glucose detecting agents, such as glucose oxidase [121,122].

Albumin is a protein found in the blood plasma. It has important biological functions, such as providing nutrients for stem cells in a lab [123,124]. Albumin is not used enough as a biomaterial in regenerative medicine. However, albumin is also used to treat patients in a number of different ways. Coating medical products with albumin can improve how they interact with the body, which can be useful for implants like bone grafts or sutures. Albumin is mostly recognised as an anti-attachment protein, but when used on implantable surfaces, its effect is the opposite: it helps stem cells to stick and grow. Albumin can be used to control the biological reaction to implanted tissue-engineering constructs because it has anti-clotting, antibacterial and anti-inflammatory properties. Another way it could be used is to mix albumin with natural or man-made materials. This creates new types of material that can be used to develop heart, brain, soft and hard tissue. New materials allow the albumin protein to be produced using a process called electrospinning. This creates new possibilities for albumin-based materials that can be used in cell therapy. Some of these technologies are already being used in hospitals, and they make good use of the fact that albumin is a natural substance that is found in the blood [123,124].

## 5. Conclusions

Further research is required to corroborate the present findings. Nevertheless, the utilisation of biocompatible external reinforcements constituted by sutures has the capacity to stimulate, guide and enhance the natural processes of biological graft remodelling and reformation to foreign materials. This may be pivotal in addressing some of the drawbacks associated with aneurysmal formation by accommodating tissue growth. The subsequent direction may involve the utilisation of resorbable suture to develop devices. The device adapted and functionally compensated for the characteristics of autograft growth, thereby ensuring a reasonable size of the autograft at 6 months. Moreover, due to its biocompatible nature, the device did not disrupt the biological process of growth or cause inflammatory damage to the wall. The material integrated with the vessel wall in a harmonious manner, inducing elastic remodelling. This remodelling is likely to have had a mitigating effect on the pressure load exerted on the vessel and may have compensated for the tendency to dilation. The remodelling is thought to have assisted somatic growth and prevented aneurysmal degeneration and stent reocclusion.

## Figures and Tables

**Table 1:** Features and uses of suture materials.

Suture Type	Strand	Unprocessed Material	Employment	Properties	Drawbacks
Polydioxanone (PDS II)	Monofilament	Polymer of paradioxanone	Used to approximate tissues over extended periods. Useful in infected tissues.	It maintains 74% of its tensile strength after two weeks and 25% after six weeks. It is absorbed by hydrolysis at 6–7 months.	The knot is not securely fastened and its handling characteristics are poor due to its stiffness and memory.
Polyglactin 910 (Vicryl)	Braided multifilament	A copolymer of glycolic and lactic acid that is coated with calcium stearate.	Less complicated to use than gut Causes less tissue reaction. More robust Used in infected wounds.	Strength is lost at the two-week mark, with 65% of strength being gone by the three-week point.	It has the potential to make a clean cut through tissue that may be friable.
Poliglecaprone 25 (Monocryl)	Monofilament	Copolymer of glycolide and epsiloncaprolacto	It is easy to handle, and it secures knots well. Minimal tissue reaction. General tissue approximation	The substance loses 30–40% of its strength after 14 days, and there is no strength remaining after 3 weeks. It is absorbed by idrolysis after 90–120 days.	The use of this product is contraindicated in cases of delayed healing.
Polyglycolic acid (Dexon, Dexon II)	Braided multifilament	A synthetic homopolymer of glycolic acid. The Dexon II has been coated with a polycarbonate coating.	This product is comparable to Vicryl. This product has a wide variety of uses, including applications in both normal and contaminated tissues. This product is ideal for use in C-sections and intestinal anastomosis.	Maintains 89% of its effectiveness after seven days, 63% after 14 days and 17% after 21 days; Is absorbed by hydrolysis within 90–120 days.	The process of breakdown is intensified in the urine and oral cavity. The uncoated form exhibits a high coefficient of friction.
Polyglycolide–trimethylene carbosnate (Maxon)	Monofilament	This product is a copolymer of glycolic acid and trimethylene.	This product has been shown to be more secure than PDS II and comparable applications, while also offering similar functionality.	The tensile strength of the material is maintained for 42–92 days. In comparison to the 64–80 days required for PDS. The product is fully absorbed by hydrolysis after six months.	In comparison to the PDSII, this product exhibits inferior handling characteristics.
Caprosyn	Monofilament	The product comprises a blend of glycolide, caprolactone, trimethylene carbonate and lactide.	This product boasts superior handling and tensile strength, along with greater knot security when compared with gut. In addition, it offers increased resistance to infection for optimal reliability.	The material displays 50–60% tensile strength at 5 days, and 0% at 3 weeks. It should be noted that the process was fully absorbed within 56 days.	Due to its short retention time, the use of this substance is restricted to plastic surgery, obstetrics and gynaecology, where rapid absorption and minimal scarring are of the essence.
Polyhydroxyalkanoates (PHAs)	Biodegradable polymers of microbial origin	4-hydroxybutyrate (4HB) polymers	Polyhydroxyalkanoates are a great choice for making stretchy medical devices in the field of biomedical engineering. They are mostly used in stitches and heart stents.	Decrease in the initial linear tensile strength of the P(3HB-co-4HB) sutures from 161 to 104.6 MPa (i.e., by 35.0%). The fractional weight-average molecular weight (Mw) exhibits a decrease. The P(3HB-co-4HB) suture showed increased elasticity and extensibility when it was broken (160% compared to the first measurement of 240%).	The suture’s circumference is smaller than PDS II’s, its knot area is smaller too and its knot area to thread diameter ratio is lower. Nevertheless, the former possesses a greater thread size and as a consequence, a considerably reduced knot diameter

Abbreviations in the text.

## Data Availability

Not applicable.
